# Factors associated with development of gastrointestinal problems in patients with scleroderma: a protocol for a systematic review

**DOI:** 10.1186/2046-4053-3-115

**Published:** 2014-10-13

**Authors:** Brian Younho Hong, Raymond Giang, Lawrence Mbuagbaw, Maggie Larche, Lehana Thabane

**Affiliations:** 1 Bachelor of Health Sciences (Honours) Program, McMaster University, Hamilton, ON, Canada; 2 Statistics for Health (Honours), University of Waterloo, Waterloo, ON, Canada; 3 Department of Clinical Epidemiology and Biostatistics, McMaster University, Hamilton, ON, Canada; 4 Biostatistics Unit, Father Sean O'Sullivan Research Centre, St. Joseph's Healthcare, Hamilton, ON, Canada; 5 Centre for Development of Best Practices in Health, Yaoundé Central Hospital, Yaoundé, Cameroon; 6 Division of Rheumatology, Departments of Medicine and Paediatrics, McMaster University, St. Joseph's Healthcare, McMaster Children's Hospital, Hamilton, ON, Canada; 7 Departments of Paediatrics and Anaesthesia, McMaster University, Hamilton, ON, Canada; 8 Centre for Evaluation of Medicine, St. Joseph's Healthcare, Hamilton, ON, Canada; 9 Population Health Research Institute, Hamilton Health Sciences, Hamilton, ON, Canada

**Keywords:** Systematic review, Scleroderma, Gastrointestinal, Risk factor

## Abstract

**Background:**

Scleroderma affects the gastrointestinal (GI) tract in 90% of all cases. Malnutrition, diarrhea, and constipation are some GI complications that can stem from scleroderma, and they contribute considerably to impairment in quality of life. Reports of haphazard clusters of high prevalence suggest that environmental exposure is a risk factor for scleroderma. However, it is largely uncertain whether the GI involvement secondary to scleroderma is influenced by these environmental factors. This study will review the association between GI involvement (unintentional weight loss, choking, early satiety, etc.) and environmental exposure in patients with scleroderma.

**Methods/design:**

Any available observational studies that report GI problems in patients with scleroderma along with the associated risk factors will be selected. We will search CINAHL, EMBASE, LILACS, MEDLINE, and Web of Science for relevant articles written in English from June 1884 to May 2014. Identified articles will be screened in duplicate, and full text for selected articles will be retrieved. Data extraction will be done in duplicate on sociodemographic characteristics of participants, diagnosis of scleroderma, diagnosis of risk of GI problem, risk factors reported, etc. Discrepancies will be resolved by consensus or by consulting a third author. We will assess the participants, methods, and intervention effects of included studies for heterogeneity. Any identified clinical or statistical heterogeneity will be explored visually or using the chi-square test. Data will be pooled statistically using the DerSimmonian and Laird random effects method if we have a measure of relative risk and its precision. Our findings will be reported according to the Meta-Analyses and Systematic Review of Observational Studies (MOOSE) guideline.

**Discussion:**

Our findings may help patients with scleroderma and health care professionals in preventing GI morbidity. Knowing that the cost of care for patients with scleroderma increases with more organ involvement, study findings can inform policy developers to identify ways to curb health care costs.

**Systematic review registration:**

PROSPERO: CRD42014010707

## Background

Scleroderma, otherwise known as systemic sclerosis, is a disease manifested by collagen overproduction affecting various organs including the gastrointestinal (GI) tract [1]. Patients with scleroderma commonly present with inflammation and fibrosis of the skin, vascular abnormalities, organ damage, and autoantibody production [2]. Diagnosis is made when these signs and corresponding symptoms are present [3]. Scleroderma is a debilitating disease that is estimated to affect approximately 16,000 Canadians, impacting women four to five times more so than men [1]. The prevalence of the disease is higher among those of African origin and varies across countries, appearing higher in North America and Australia than in Europe and Japan [4]. Scleroderma is associated with debilitating morbidity such as reduction in mobility and depression [5]. Patients with this disorder have 30.8% mortality rate, although this number can vary depending on gender and what visceral organs are involved [6]. Currently, there are no effective treatments to combat scleroderma, partly because its pathogenesis remains unclear [7]. Consequently, much attention has shifted towards predicting complications secondary to scleroderma and to manage them appropriately [8]. The underlying rationale is that it is easier to treat the stage before these complications arise [8].

The GI tract issues are involved in 90% of all scleroderma patients and contribute considerably to impairment in quality of life [9-11]. Malnutrition, nausea, vomiting, diarrhea, and constipation are some GI complications that can stem from scleroderma [9]. Reports of nonlinear clusters of high prevalence suggest that environmental exposure is a risk factor for scleroderma [11]. For example, silica along with silicone and epoxy resins have been associated with increased risk of scleroderma [11]. It is largely uncertain, however, whether the GI involvement secondary to scleroderma is influenced by these environmental factors. This study will review the association between GI involvement and environmental exposure in patients with scleroderma. These findings will help patients with scleroderma and health care professionals in preventing the GI morbidity. In Canada, the average annual economic burden of scleroderma is estimated to be $18,453 per patient [12]. Knowing that the cost to care for patients with scleroderma increases with more organ involvement, the study findings can inform policy developers to identify ways to curb health care costs [12].

The overall purpose of this systematic review is to determine the factors associated with development of GI problems in patients with scleroderma. We will look for risk factors for GI problems in patients with scleroderma. These risk factors include silica, silicone implantation and rupture, antacid, solvents, epoxy resins, welding fumes, anorexigens, pentazocine, bromocriptine, l-tryptophan, pesticides, constant hand/arm vibration, and alcohol [13-18]. We will consider additional risk factors, such as intestinal microbiota and food, in the event that we find any studies that report on them. We will identify and report other predictors of and factors associated with development of GI problems in scleroderma patients.

## Methods/design

### Inclusion/exclusion criteria

#### Types of studies

Any available observational studies that report on GI problems in patients with scleroderma along with the associated risk factors will be selected for the review.

They must have the following criteria:

1. Patients with a formal diagnosis of scleroderma according to 1980 and 2013 criteria for the diagnosis of systemic sclerosis by the American College of Rheumatology [19,20].

2. Clearly reported GI problems.

3. Specific risk factors for GI problems will be studied.

#### Study settings

Literature from any geographic or socioeconomic setting will be used.

#### Types of participants

All participants will be included provided they have scleroderma and are assessed for risk factors for GI problems.

#### Intervention/exposure

We will look for risk factors for GI problems in patients with scleroderma. These risk factors may include silica, silicone implantation and rupture, antacid, solvents, epoxy resins, welding fumes, anorexigens, pentazocine, bromocriptine, l-tryptophan, pesticides, constant hand/arm vibration, and alcohol [13-18]. We will consider additional risk factors, such as intestinal microbiota and food, in the event that we find any studies that report on them. We will identify and report other predictors of and factors associated with development of GI problems in scleroderma patients.

### Search strategy for identification of studies

We shall attempt to identify all studies relevant to the GI problems in patients with scleroderma written in English from June 1884 to May 2014. June 1884 was chosen because it is the earliest entry of scleroderma in available databases.

#### Electronic searches

We will search Cumulative Index to Nursing and Allied Health Literature (CINAHL), Excerpta Medica database (EMBASE), Latin American and Caribbean Health Sciences Literature (LILACS,) Medical Literature Analysis and Retrieval System Online (MEDLINE), and Web of Science for relevant articles written in English from June 1884 to April 2014. We will use the subject term services of the different databases. The following search terms and their medical subject heading (MeSH) equivalents will be used: scleroderma, risk factors, gastrointestinal, unintentional weight loss, poor appetite, dysphagia, acid reflux, choking, early satiety, bloating, nausea, vomiting, constipation, diarrhea, antibiotic use, steatorrhea, fecal incontinence, parenteral nutrition, and the above listed risk factors. These terms will be used in varying combinations. Table 1 is a proposed search strategy for MEDLINE via Ovid.

**Table 1:** Proposed search strategy of ovid

**Database: Ovid MEDLINE(R)**	
**Search strategy**	
1	scleroderma.mp.
2	systemic sclerosis.mp.
3	exp scleroderma, systemic/
4	exp scleroderma, localized/
5	or/1-4
6	risk.mp.
7	exp risk/
8	((factor$ or feature$ or aspect$ or characteristic$) adj4 (risk)).mp.
9	exp silicon dioxide/
10	exp silicones/
11	exp antacids/
12	exp solvents/
13	organic solvent$.mp.
14	exp epoxy resins/
15	welding fume$ or welding gas*.mp.
16	exp anorexigenic drugs/
17	exp pentazocine/
18	exp bromocriptine/
19	exp tryptophan/
20	exp pesticides/
21	exp vibration/
22	hand vibration or arm vibration.mp.
23	exp alcohol drinking/
24	or/6-23
25	exp gastrointestinal tract/
26	exp gastrointestinal diseases/
27	((problem$ or issue$ or concern$ or complication$) adj3 (alimentary canal or alimentary tract or digestive tube or digestive tract or GI tract)).mp.
28	exp weight loss/
29	uninten** weight loss.mp.
30	((uninten**) adj3 (weight loss)).mp.
31	exp appetite/
32	poor appetite or limited appetite or reduced appetite or sparse appetite.mp. 34. ((poor or limited or reduced or sparse) adj3 (appetite)).mp.
35	exp deglutition disorders/
36	dysphagia or swallowing disorders.mp.
37	exp gastroesophageal reflux/
38	acid reflux.mp.
39	exp colonic pseudo-obstruction/
40	exp duodenal obstruction/
41	exp intestinal obstruction/
42	exp intestinal pseudo-obstruction/
43	chok*.mp.
44	early satiety.mp.
45	bloat* or disten* or bulg* or enlarg* or expan*.mp.
46	exp nausea/
47	exp vomiting/
48	exp constipation/
49	exp diarrhea/
50	((use or prescri** or administ**) adj2 (antibiotic$ or antibacterial$ or antimicrobial$)).mp.
51	exp steatorrhea/
52	exp fecal incontinence/
53	exp parenteral nutrition/
54	or/25-53
55	5 and 24 and 54

#### Reference lists

We will manually check the reference list of all the studies identified by the above search strategy. Relevant studies shall be read and included in the review when deemed appropriate. We will also check the reference list of any previous reviews on GI problems secondary to scleroderma for any relevant articles, which could be included in the review.

#### Gray literature

We will attempt to contact authors, experts in the field, research organizations, and conference websites for any relevant material.

### Data collection and analysis

#### Screening

Two authors (BYH and RJ), working independently, will screen all citations and abstracts resulting from the search strategy to identify eligible papers. The full text of eligible articles will be obtained and assessed using a pre-designed eligibility form based on the inclusion criteria. Eligible studies will be included in the review. Disagreements will be resolved by discussion or by seeking the opinion of a third party (LT/ML).

Authors will be contacted to clarify missing or unclear information. Ineligible studies will be excluded from the review, and the reason for exclusion will be stated in a table.

#### Data extraction

The two authors (BYH and RJ) will independently extract data from the studies using a pre-established data extraction form. Data will be extracted on sociodemographic characteristics of participants (age, gender, occupation, residence), diagnosis of scleroderma, diagnosis of risk of GI problem, risk factors reported, study design, study setting, sample size, lost to follow-up/missing data, and strength of association between risk factors and outcome, if reported.

In order to pool the risk factors for GI problems among the participants with scleroderma, we will extract the numbers with each identified risk factor among those with GI problems and the numbers without the risk factor so we can construct two-by-two tables and use them for meta-analyses. If only the odds ratios are reported with a measure of precision (confidence interval or standard error), we will use this in the meta-analysis.

#### Assessment of methodological quality

The two authors (BYH and RJ) will independently assess the methodological quality of included studies using the Newcastle-Ottawa Scale [21]. We will be assessing publication bias using funnel plots along with other biases such as reporting bias, performance bias, and detection bias.

### Analyses and reporting

#### Data synthesis and assessment/investigation of heterogeneity

We will only pool the results if there is a measure of relative risk (risk ratio, odds ratio, hazard ratio) and its precision (95% confidence intervals, standard error, variance). We will assess the participants, methods, and intervention effects of included studies for heterogeneity. This will enable us to determine whether results can be pooled across studies. Any identified clinical or statistical heterogeneity will be explored visually or using the chi-square test. Possible sources of heterogeneity include the population studied, the severity of the condition, the severity of the exposure, duration of follow-up, etc.

#### Statistical analysis

Comparison of risk will be made against scleroderma patients with the risk of GI concerns who have not developed GI problems. Data will be pooled statistically using the DerSimmonian and Laird random effects method where appropriate [22]. The results will be expressed as odds ratios with 95% confidence interval and *p* values. Between-study heterogeneity will be tested using Cochran's Q and the *I*^2^ statistic. Stratified analysis of children versus adults and inpatients versus outpatients may be conducted. Data will be analyzed using Review Manager version 5.2.

#### Sensitivity analyses

A sensitivity analysis will be performed to explore how robust the results are to variations in methodological quality.

#### Presenting and reporting of results

The analysis and reporting of the review will be done according to the Meta-Analyses and Systematic Review of Observational Studies (MOOSE) guideline [23]. We will use a flow diagram to summarize the selection process of the studies. Agreement between data abstractors will be assessed using the kappa statistic [24]. A full list of excluded studies with reasons for exclusion will be reported.

## Discussion

GI manifestations secondary to scleroderma are multifaceted depending on the parts of the alimentary tract involved [11]. Severe GI problems are associated with higher rates of mortality [25]. Studies report that 90% of scleroderma patients experience GI problems underscoring the quality of life concerns associated with them [11]. The information obtained from the study will be important for patients, clinicians, and policy developers. By identifying and reporting predictors of and factors associated with GI problems in scleroderma patients along with early biomarkers that can predict the future severity of the disease, this study will help clinicians to improve risk assessment. The resultant earlier and more effective diagnosis can aid patients in reducing the GI morbidity. By identifying ways to prevent GI morbidity, this study can curb the cost of health care in helping scleroderma patients.

### Ethics and dissemination

The results of this paper will be disseminated as peer-reviewed publications, at conferences and at clinical rounds.

## Abbreviations

CINAHL: Cumulative Index to Nursing and Allied Health Literature; EMBASE: Excerpta Medica database; GI: gastrointestinal; LILACS: Latin American and Caribbean Health Sciences Literature; MEDLINE: Medical Literature Analysis and Retrieval System Online; MOOSE: Meta-Analyses of Observational Studies in Epidemiology.

## Competing interests

The authors declare that they have no competing interests.

## Authors’ contributions

BYH, ML, and LT conceived of the study, revised the research question, and provided content to the design. RG and LM revised the research question and provided content to the design. All authors read and approved the final version of the manuscript.
